# The potential application of biochar and salicylic acid to alleviate salt stress in soybean (*Glycine max* L.)

**DOI:** 10.1016/j.heliyon.2024.e26677

**Published:** 2024-02-19

**Authors:** Mohammad Mehdi Alizadeh, Mahyar Gerami, Parastoo Majidian, Hamid Reza Ghorbani

**Affiliations:** a Department of Biology, Sana Institute of Higher Education, Sari, Iran; b Crop and Horticultural Science Research Department, Mazandaran Agricultural and Natural Resources Research and Education Center, Agricultural Research, Education and Extension Organization (AREEO), Sari, Iran

**Keywords:** Soybean, Biochar, Salicylic acid, Physiological and morphological traits

## Abstract

Salt stress has been one of the major contributor which affect soybean seed germination, its establishment, growth, and physiology stages. Utilization of strategies such as soil amendment and elicitors are of significant importance to reduce the disadvantageous effects of salt stress. In this regard, the objectives of the present study were to evaluate the effect of biochar and salicylic acid on morphological and physiological properties of soybean subjected to salinity. The first experiment was carried out based on completely randomized design with three replications including 11 soybean cultivars such as Williams, Saba, Kowsar, Tapor, Sari, Telar, Caspian, Nekador, Amir, Katol and Sahar and various levels of salinity such as 0, 2, 4, 6 dS/m of NaCl. The second experiment was performed as factorial design in a randomized complete block design with three replications consisting of treatments of biochar (0, 5 and 10 WP), salicylic acid (0, 0.5 and 1 mM), and NaCl (0, 2.5, 5, 7.5 dS/m). With respect to seed germination result, various concentrations of salt stress showed negative impact not only on all studied traits, but also varied among soybean cultivars indicating Amir cultivar as the best salt tolerant soybean genotype among others. In addition, our data exhibited that the interaction effect of biochar and salicylic acid on salt treated soybean plant were positively significant on some morphological traits such as leaf area, shoot dry/fresh weight, total dry/fresh weight and physiological attributes including chlorophyll *a*, flavonoid, proline contents, catalase and peroxidase activities. Moreover, the resultant data showed that the combination treatment of 5 and 10 WP of biochar and 1 mM of salicylic acid caused increase of the aforementioned parameters in order to improve their performance subjected to higher concentration of salinity. In final, it was concluded that the coupled application of biochar alongside salicylic acid was recommended as proficient strategy to mitigate the injurious influences of salt stress in soybean or other probable crops.

## 1 Introduction

Nowadays, environmental stresses such as abiotic and biotic stresses have been became the center of concern for various researchers worldwide since they have adverse negative effects on plant life cycle from seed germination up to crop survival and its performance [1]. Among different environmental stresses, salt stress is one of important limiting factor of all vegetative and reproductive growth stages of plant specially in arid and semi-arid regions [2]. It has reported that more than 45 million hectares of agricultural lands have been exposed to salinity stress which have resulted in conversion of these lands into arable lands or bring highly-cost of their reform [3].

In case of salinity, numerous studies have been performed to evaluate the interaction effect of salinity on various aspects of crops resulting the significant negative effect of salinity on crop growth and development stages including nutritional disorder [4,5], ion toxicity [6], oxidative stress [7,8], metabolic processes changes [9], and various disorders in biochemical, physiological and molecular mechanisms. Thus, assessment of physiological and biochemical mechanisms of plants under salt stress could help achieve improvement in agricultural production through introduction of tolerant/resistant cultivars or applicable tools to reduce destructive effects of salt stress following by increase plant's performance.

Plants indicate different reactions facing salt stress based on the type of plant, the exposure dose of salt concentration and the time occurrence. Among various plants species, legume crops specially soybean is relatively sensitive to salt stress resulting in decrease its yield up to 40% rely on salinity level [10]. In soybean, the higher devastating effects of salt stress is observed in different growth stages such as seed germination, seedling and developmental stages as well as nodulation process [11]. For example, one study showed decrease in AOX respiration, root and shoot K+/N+ ratio, leaf area and water use efficiency under salt stress [12]. On the other study, the seed germination of soybean was delayed at lower level of salt stress (0.05 and 0.1% of NaCl), and it was reduced at higher concentration of salinity [13].

In this case, utilization of elicitors to alleviate the harmful effects of salt stress would be of great importance in order to combat disturbances of salt stress in plants. Salicylic acid (SA) is a phyto-hormone cause increase of tolerance to abiotic stresses like salt and drought through photosynthesis regulation, stomata opening, cell growth, decrease in ion leakage and disturbance caused by oxidative stress [14]. In addition, SA leads to increment of relative water content of leaves and osmotic pressure regulation in plants under environmental stresses [15]. Few studies were performed on the impacts of SA on growth stages and physiological processes of soybean under salt stress. For instance, the one study reported that the utilization of SA enhanced the nutritional status such as calcium and potassium contents in soybean under salt stress emphasizing on the relieve of toxicity signs of salinity by simultaneous application of selenium and salicylic acid [16]. On the other research, the protein percentage and yield of soybean improved at different stages of seed development under salt stress through increment the contents of amino acids such as isoleucine, leucine, lysine, methionine, valine, alanine, aspartic acid, glutamic acid, glycine and serine contents [17]. In the other similar research, the effects of salicylic acid and jasmonate acid on oil accumulation and fatty acid composition of soybean seed were evaluated under salt stress resulting the improved oil quality (by reduction of oleic acid and increase of linoleic acid and linolenic acid contents) by foliar application of SA [18].

According to salt stress disadvantageous, this point is extremely important that the formation of saline-alkali soils can obtain from strong evaporation in arid and semi-arid areas which cause crops suffer the dual detrimental effects of drought and salinity stresses. Thus, it is necessary to mitigate and compensate the unpleasant effects of these abiotic stresses which will be considerable for agricultural sustainability and food security. In this regard, biochar is a promising agronomic measure which is prepared from waste of organic materials with the capability of improving harsh soil environmental situations [19]. Indeed, biochar is able to decline destructive damages of saline stress during crop development. Biochar is a type of coal obtained from plant biomass and agricultural waste which it burns at low/without level of oxygen at 300–1000 °C [20]. This natural material disintegrates at low speed resulting the decrease of greenhouse gases such as carbon dioxide and methane [21]. Moreover, biochar includes significant contents of calcium, magnesium and inorganic carbonates which are valuable for crops [22]. In total, biochar can develop the physiochemical conditions for plants and microorganism by changing soil properties including bulk density, pH, cation exchange capacity, soil structure, and water retention capacity [23].

However, there are limited researches on the role of biochar in soybean crop under salt stress. Therefore, the objective of this study is to assess the effects of salicylic acid accompanying by biochar on morphological and physiological properties of soybean in response to salt stress conditions.

## 2 Materials and methods

This project was performed in two stages at Baye-Kola Agricultural Research Station of Mazandaran Agricultural and Natural Resources Research and Education Center and Sana Institute of Higher Education of Sari in 2021–2022. In the first experiment, the primary salt tolerance was carried out among 11 soybean cultivars based on completely randomized design with three replications at four levels of salts (0, 2, 4, 6 dS/m). The soybean cultivars had different maturity groups including early-mature such as (Williams, Saba and Kowsar) appropriate for Ardebil province, medium-mature suitable for Mazandaran province such as (Tapor, Sari, Telar, Caspian and Nekador) and medium-mature including (Amir, Katol, Sahar) compatible for Golestan province.

Firstly, the soybean seeds were disinfected by 1% hypochlorite sodium for 5 min and then rinsed with distilled water. Then, 20 seeds were placed on filter paper at 10 cm-diameter of Petri dish including different concentrations of NaCl (0, 2, 4 and 6 dS/m) with three replications. The plates were transferred into growth chamber with humidity (65%), temperature (25 °C) and light condition of 16h darkness and 8h lightness in order to incubate and activate seeds for starting germination. The evaluation of seed germination was performed after 24 h and continued until all seeds were able to germinate. When the rootlet length was about 2 mm or more, 10 ml of NaCl solution was added to each Petri dish [24].

After selection of salt tolerant cultivar, the second experiment was carried out as factorial design in a randomized complete block design with three replications in order to evaluate the interaction effects of SA (0, 0.5 and 1 mM), biochar (0, 5, and 10 WP) and salt (0, 2.5, 5, 7.5 dS/m) on selected soybean cultivar (Amir) attributes. In this case, 10 soybean seeds were disinfected by sodium hypochlorite (1%) for 1 min and then rinsed with distilled water. Then, the plastic pots (size of 30 × 23 × 26 cm) were filled with the same proportion of soil, sand and manure as well as various treatments of biochar following by seed cultivation at 3–5 cm of soil depth. Plants were kept under the natural light of day with supplementary light that was kept 13 h photoperiod with irradiance at plant level of 900–1200 μmol m^2^s^1^ (PAR). Temperature and relative air humidity were 27 ± 3^°^С and 60 ± 5% respectively. After plant stability and establishment, they were thinned to six plants. The salinity and SA foliar application were accomplished when plants were 4 weeks old (Four nodes on the main stem with fully developed leaves beginning with the unifoliate nodes). The salinity treatments were carried out every three days based on 75% of field capacity. In order to prevent the side effects of lack of nutritional elements, the pots watered by Hoagland solution without chlorine and sodium. At the end of experiment, electric conductivity of each pot was measured by using a digital conductivity meter (Inolab Model, Weilheim, Germany) to estimate final concentration of NaCl levels.

### 2.1 Seed germination parameters

The germination factors such as rootlet and shootlet length, dry weight of rootlet and shootlet, total dry weight, germination percentage and germination rate were measured. The length of rootlet and shootlet was measured by kolis and the germination rate was calculated by formula (1):

(1) $$GR=\sum \frac{Li}{Di}$$

Where the GR is growth rate, Li is the number of seed germinated in ith day, and Di is the number of days from the first day of cultivation. Moreover, the germination percentage is calculated by formula (2), where the GP is germination percentage, N is the total germinated seed, and M is the total cultivated seed.

(2) $$GP=\frac{\left(N*100\right)}{M}$$

After 7 days, the fresh weight of each sample was measured by exact weigh and then samples were wrapped in the aluminum foil and kept in oven at 70 °C for 72h to get dried. Finally, dry weight was determined by weighing the dried roots and shoots using precision balance.

### 2.2 Plant growth factors

After 6 weeks old of soybean plants, three to five samples were collected to measure morphological parameters such as root and shoot fresh weight, total fresh weight, and leaf area (LA). Then, plants individually were wrapped in brown paper bag and kept in oven at 70 °C for 72 h following by determination of dry weight of each sample using precision scale.

### 2.3 Physiological assays

#### 2.3.1 Chlorophyll and carotenoid content

To measure carotenoid and chlorophyll contents, 0.5 g of each sample leaf were homogenized using 5 ml of acetone (80%) following by centrifugation of 13000 rpm/min and 4 °C for 15 min. Then, optical absorbance of chlorophyll *a*, chlorophyll *b* and carotenoid was measured at wavelengths of 663, 645 and 490 nm, respectively using spectrophotometry (Shimadzu uv 180) [25]. The following equations (3), (4), (5) and (6) were used to measure the amount of pigment in chlorophyll *a*, chlorophyll *b*, chlorophyll (a + b) and carotenoid content, respectively (Li et al., 2006).

(3) Chla(mg/gFW)=12.25×A_(663.2)−2.79×A_(646.8)

(4) Chlb(mg/gFW)=21.51×A_(646.8)−5.1×A_(663.2)

(5) Chl(a+b)=Chla+Chlb

(6) Carotenoid(mg/gFW)=(1000×A_(470)–1.8×Chla–85.52×Chlb)/198

#### 2.3.2 Soluble sugar content

The amount of soluble sugar was measured according to the Antron Method by spectrophotometry (Shimadzu uv 180) at 620 nm wavelength [26]. About 0.1 g of fresh sample were crushed with 5 mM of 80% ethanol in a mortar and were placed in bain-marie for 15 min. The extracted ethanol containing soluble carbohydrate was separated and the previous procedure was repeated for the lower part of the extraction for four times. In order to evaporate the ethanol, the obtained extraction was placed at temperature of 70 °C. Then, the extraction was mixed with chlorophorm (1:5) and the upper part was centrifuged at 10,000 rpm for 10 min. The transparent part of the solution was separated for measuring soluble carbohydrate. The mixture was heated at 100 °C for 1 min and absorbance was read at 620 nm.

#### 2.3.3 Proline determination

Proline accumulation was determined according to proline's reaction with ninhydrin [27]. Approximately 0.5g of fresh or frozen plant material was homogenized in 10 ml of 3% aqueous sulfosalicyclic acid and filtered. For proline colorimetric determinations, two ml of filtrate was mixed with 2 ml acid-ninhydrin and 2 ml of glacial acetic acid and then incubated at 100 °C for 1 h. The reaction was arrested in iced bath and the cromophore was extracted with 4 ml toluene. Its absorbance was determined at 520 nm in spectrophotometer (Shimadzu UV 180).

#### 2.3.4 Total flavonoid content

The total flavonoid content was measured by a colorimetric assay [28]. One hundred μl of the extract was added to 1711 μl of ethanol 31%, 151 μl of sodium nitrate 0.5 mM and 151 μl of aluminum chloride 0.3 mM. In 5 min, 111 μl of 1 mM sodium hydroxide was added to the mixture. Then, after 11–15 min, the absorbance was determined at 510 nm versus a blank. Total flavonoids content of the extract was expressed as mg catechin equivalents per gram of sample.

#### 2.3.5 Free radical scavenging activity

DPPH radical scavenging activity was measured according to the method of [29]. The absorbance of samples was carried out at 517 nm in order to determine he reduction of DPPH radicals. The percentage of radical scavenging activity was calculated as a percentage of DPPH discoloration using the following equation (7):

(7) DPPHradicalscavenging%=[(A0–A1)/A0]×100

Where A0 is the absorbance of the DPPH solution and A1 is the absorbance of the sample.

#### 2.3.6 Peroxidase (POD) and catalase activity

POD activity was determined by measurement of the absorbance at 470 nm using guaiacol and H_2_O_2_ as substrate by spectrophotometer (Shimadzu UV 180). Briefly, 490 μl of 45 mM guaiacol and 490 μl of 225 mM H_2_O_2_ were mixed in 50 mM phosphate buffer (pH: 7.0) at 4 °C and the reaction was started by addition of 20 μl of POD solution [30].

To measure catalase activity, the enzyme extract (20 μl) was added to the reaction mixture containing 3 ml of H_2_O_2_ and 50 mM phosphate buffer (pH 7.0) and the OD changes was measured at 240 nm wavelength, the time taken for decrease in the absorbance from 0.45 to 0.4 is noted as ΔT [31].

### 2.4 Statistical analysis

Analysis of variance was performed using SAS version 9.1 (SAS Institute Inc., Cary, NC, USA). The mean comparison of each treatment were analyzed using the LSD test at the 5% probability level.

## 3 Results

### 3.1 Effect of salt stress on soybean cultivars based on seed germination parameters

**Fig. 1:**
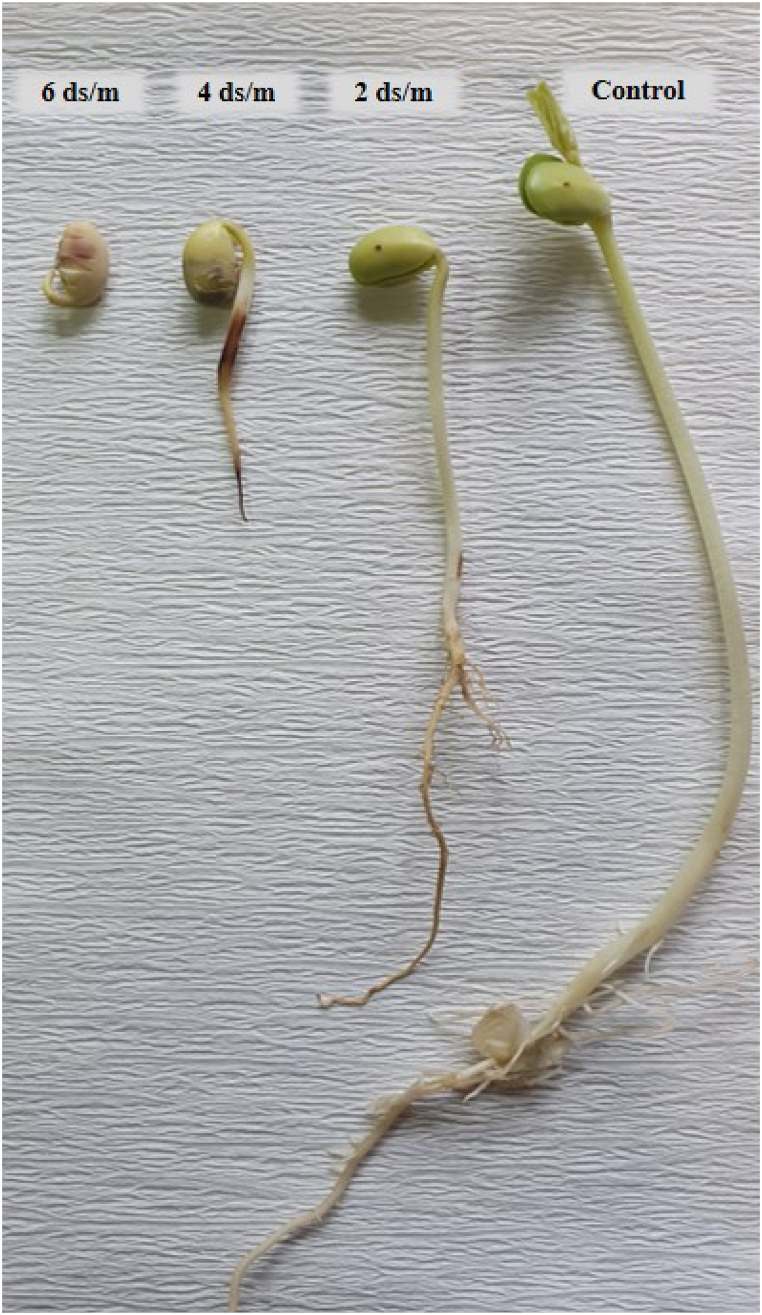
The effect of different concentration of salinity on seed germination of Amir cultivar after 7 days of germination. From right side, there is control sample (0), 2, 4 and 6 dS/m of NaCl.

The mean comparison of the interaction effect of salt stress and soybean cultivar on different seed germination traits showed that the highest and lowest amount of each parameter was observed in 0 and 6 dS/m of salinity, respectively. By increase of salinity levels, the amount of each trait decreased emphasizing the highest reduction of traits at 6 dS/m of salt stress (Fig. 1).

#### 3.1.1 Rootlet and shootlet length

With increase of salinity, the rootlet and shoot let of each soybean cultivar decreased significantly (Table 1). The highest amount of rootlet and shootlet length was 143.33 and 133.33 mm which was observed in Katol cultivar without salinity. The lowest amount of rootlet and shootlet length were indicated in Sahar cultivar (9.00 and 4.66 mm, respectively) and Williams (10.66 and 6.33 mm) at 6 dS/m of salinity (Table 1). Although, the Amir cultivar didn't show the highest amount of these traits at 0 dS/m of salinity level, it could exhibit the highest amount of rootlet (90.00 and 51.66 at 2 and 4 dS/m of salinity, respectively) and shootlet (53.33 and 36.66 mm at 2 and 4 dS/m of salinity, respectively) (Table 1). This result implies the appropriate salt tolerance of Amir cultivar in relative increment of salinity. At 6 dS/m of salinity, the maximum amount of rootlet length was related to Telar (18.00 mm), Nekador (17.00 mm) and Saba (16.00 mm) and the maximum amount of shootlet length was 11.00 mm which was observed in Saba and Caspian cultivars (Table 1).

**Table 1:** Mean comparison of interaction effect of variety and salinity treatment on germination traits.

Treatment	Germination rate	Total dry weight	Shoot dry weight	Root dry weight	Shoot length	Root length
S0						
**Wiliams**	^a^ 10.00	^l-n^ 0.157	^i-m^ 0.113	^op^ 0.043	^k-n^ 28.33	^l-p^ 30.00
**Saba**	^a^ 10.00	^a^ 0.507	^b^ 0.312	^a^ 0.216	^fg^ 65.00	^b-d^ 101.66
**Tapoor**	^a^ 10.00	^b^ 0.454	^c^ 0.283	^b^ 0.170	^de^ 84.33	^d-g^ 85.00
**Sari**	^f-j^ 6.33	^f-h^ 0.240	^fg^ 0.168	^k^ 0.071	^ef^ 76.67	^d-f^ 90.00
**Amir**	^a-d^ 8.33	^a^ 0.503	^a^ 0.361	^cd^ 0.141	^ab^ 120.00	^bc^ 110.00
**Caspian**	^a^ 10.00	^d^ 0.366	^de^ 0.249	^e-g^ 0.116	^dc^ 93.33	^b^ 120.00
**kosar**	^a^ 10.00	^c^ 0.412	^cd^ 0.261	^c^ 0.151	^bc^ 113.33	^bc^ 110.00
**Katol**	^a^ 10.00	^c^ 0.418	^de^ 0.241	^b^ 0.177	^a^ 133.33	^a^ 143.33
**Sahar**	^a^ 10.00	^ij^ 0.203	^g-i^ 0.140	^k-n^ 0.062	^cd^ 100.00	^c-e^ 96.66
**Telar**	^a^ 10.00	^de^ 0.344	^e^ 0.226	^ef^ 0.118	^ab^ 123.33	^g-i^ 70.00
**S2**						
**Nekador**	^a^ 10.00	^d^ 0.350	^f^ 0.182	^b^ 0.167	^a-c^ 116.66	^b-d^ 103.33
**Wiliams**	^i-k^ 5.00	^no^ 0.129	^l-o^ 0.095	^p-r^ 0.033	^k-m^ 30.00	^l-o^ 31.66
**Saba**	^ab^ 9.50	^gf^ 0.269	^f-h^ 0.158	^f-h^ 0.111	^h-k^ 45.00	^d-g^ 86.66
**Tapoor**	^b-e^ 8.111	^h-j^ 0.207	^j-n^ 0.107	^hi^ 0.100	^h-l^ 43.33	^kl^ 39.00
**Sari**	^g-j^ 6.111	^l-n^ 0.153	^k-n^ 0.106	^op^ 0.046	^h-k^ 45.00	^k-m^ 35.00
**Amir**	^a-c^ 8.611	^e^ 0.312	^f^ 0.176	^cd^ 0.136	^gh^ 53.33	^d-f^ 90.00
**Caspian**	^d-i^ 6.694	^kl^ 0.167	^l-o^ 0.090	^k^ 0.077	^h-m^ 35.66	^hij^ 65.33
**kosar**	^b-f^ 7.944	^gh^ 0.237	^hi^ 0.135	^g-i^ 0.101	^h-l^ 43.33	^f-h^ 71.66
**Katol**	^h-j^ 5.667	^h-j^ 0.208	^i-l^ 0.114	^ij^ 0.094	^g-j^ 50.00	^e-h^ 81.66
**Sahar**	^g-j^ 5.944	^no^ 0.128	^o-q^ 0.077	^m-o^ 0.051	^k-n^ 27.33	^kl^ 38.33
**Telar**	^i-k^ 5.194	^h-j^ 0.227	^h-k^ 0.132	^hi^ 0.095	^h-k^ 45.00	^f-h^ 71.66
**Nekador**	^e-j^ 6.444	^hi^ 0.233	^h-j^ 0.134	^hi^ 0.098	^g-i^ 51.66	^e-h^ 80.00

Treatment	Germination rate	Total dry weight	Shoot dry weight	Root dry weight	Shoot length	Root length
S4						
**Wiliams**	^l^ 2.972	^p-r^ 0.085	^q-t^ 0.052	^p-r^ 0.033	^k-m^ 31.00	^kl^ 37.66
**Saba**	^a-c^ 8.555	^l-n^ 0.145	^k-n^ 0.105	^o-q^ 0.040	^m-p^ 20.00	^kl^ 37.66
**Tapoor**	^b-f^ 7.972	^h-j^ 0.208	^i-k^ 0.130	^jk^ 0.078	^j-m^ 32.66	^k-m^ 35.00
**Sari**	^kl^ 3.611	^pq^ 0.094	^q-t^ 0.055	^o-q^ 0.0387	^k-n^ 27.66	^kl^ 37.33
**Amir**	^c-g^ 7.639	^f^ 0.270	^hi^ 0.140	^de^ 0.130	^h-m^ 36.66	^i-k^ 51.66
**Caspian**	^jk^ 4.861	^l-o^ 0.135	^m-p^ 0.086	^n-p^ 0.049	^i-m^ 35.00	^kl^ 43.33
**Kosar**	^c-h^ 7.111	^jk^ 0.200	^h-k^ 0.131	^kl^ 0.068	^i-m^ 35.00	^j-l^ 48.33
**Katol**	^f-j^ 6.333	^l-o^ 0.139	^n-p^ 0.086	^l-o^ 0.053	^m-p^ 21.66	^k-n^ 32.66
**Sahar**	^g-j^ 5.927	^p-r^ 0.085	^p-s^ 0.060	^q-s^ 0.025	^k-m^ 29.33	^kl^ 38.00
**Telar**	^h-j^ 5.805	^m-o^ 0.131	^l-o^ 0.089	^op^ 0.043	^l-o^ 26.66	^l-o^ 31.66
**Nekador**	^e-j^ 6.555	^lm^ 0.163	^l-o^ 0.097	^k-m^ 0.065	^k-m^ 30.00	^i-k^ 51.66
**S6**						
**Wiliams**	^m^ 0.000	^u^ 0.019	^uv^ 0.016	^t^ 0.003	^p^ 6.33	^q^ 10.66
**Saba**	^m^ 0.583	^op^ 0.106	^p-r^ 0.061	^op^ 0.045	^n-p^ 11.00	^m-q^ 16.00
**Tapoor**	^m^ 0.000	^s-u^ 0.051	^r-u^ 0.036	^st^ 0.015	^p^ 5.66	^q^ 10.66
**Sari**	^m^ 0.000	^q-s^ 0.067	^q-t^ 0.050	^r-t^ 0.018	^p^ 6.00	^n-q^ 14.66
**Amir**	^m^ 0.416	^q-s^ 0.071	^r-t^ 0.046	^q-s^ 0.025	^p^ 5.00	^o-q^ 13.00
**Caspian**	^m^ 0.327	^pq^ 0.087	^q-t^ 0.050	^o-q^ 0.037	^n-p^ 11.00	^o-q^ 13.00
**Kosar**	^m^ 0.600	^r-t^ 0.053	^s-v^ 0.033	^r-t^ 0.018	^n-p^ 10.66	^o-q^ 13.00
**Katol**	^m^ 0.766	^s-u^ 0.044	^t-v^ 0.029	^st^ 0.0145	^p^ 6.66	^pq^ 12.33
**Sahar**	^m^ 0.583	^u^ 0.018	^v^ 0.008	^st^ 0.009	^p^ 4.66	^q^ 9.00
**Telar**	^m^ 0.133	^tu^ 0.032	^uv^ 0.016	^st^ 0.016	^p^ 8.00	^m-q^ 18.00
**Nekador**	^m^ 0.066	^op^ 0.106	^p-r^ 0.061	^op^ 0.045	^op^ 9.00	^m-q^ 17.00

The means in each column that have at least one similar letter, are not significantly different. S0 and S2 are related to salt treatments as 0 and 2 dS/m of salinity, respectively.

The means in each column that have at least one similar letter, are not significantly different. S4 and S6 are related to salt treatments as 4 and 6 dS/m of salinity, respectively.

#### 3.1.2 Rootlet and shootlet dry weight

The highest amount of rootlet dry weight was belonged to Saba cultivar (0.216 g) and the lowest amount of this trait was belonged to Williams and Sahar (0.003 and 0.009 g, respectively) without salinity (Table 1). In addition, the highest amount of rootlet dry weight was obtained Amir cultivar as 13.62 and 13.00 g at 2 and 4 dS/m of salinity, respectively. At 6 dS/m of salinity, the Saba cultivar indicated 0.045 g of rootlet dry weight (Table 1). Moreover, Amir cultivar showed the maximum amount of shoot dry weight and total weight which were (0.361–0.503 g) at 0 dS/m, (0.171–0.312 g) at 2 dS/m and (0.140–0.270 g) at 4 dS/m of salinity. At 6 dS/m of salinity, the Saba cultivar indicated the highest amount of shoot dry weight (0.061 g) and total weight (0.106 g). Also, the lowest amount shoot dry weight and total dry weight were belonged to Williams (0.016 and 0.008 g) and Sahar (0.019 and 0.018) cultivars (Table 1).

#### 3.1.3 Seed germination rate

At 0 dS/m of salinity, all cultivars showed the highest rate of seed germination except Sari cultivar and the lowest rate of this parameter was obtained in cultivars of Williams, Tapor and Sari (Table 1). The highest rate of seed germination was indicated in Saba and Amir (9.50 and 8.61) at 2 dS/m of NaCl, Saba and Tapor (8.55 and 7.92) at 4 dS/m of NaCl and Katol (0.76) at 6 dS/m of NaCl (Table 1).

### 3.2 Effect of biochar and salicylic acid on morphological traits of soybean under salt stress

**Fig. 2:**
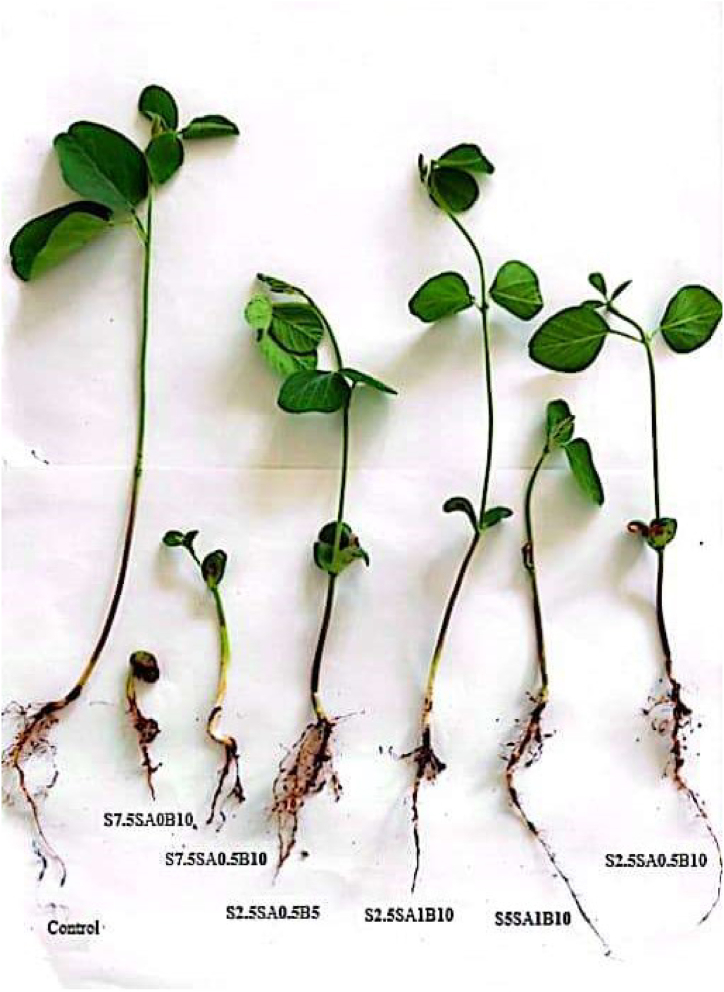
The effect of biochar, salicylic acid and salt stress on growth parameters of Amir cultivar after six weeks of germination. S, SA and B are abbreviated of salt, salicylic acid and biochar.

The leaf area, total fresh/dry weight, shoot fresh/dry weight were significantly affected by application of singular/coupled biochar, salicylic acid under salt stress (Fig. 2). The diverse biochar concentrations separately had no positive effect of the traits studied without salinity condition, nevertheless, the amount of all morphological parameters were increased by application of biochar under salinity conditions (Fig. 2).

**Fig. 3:**
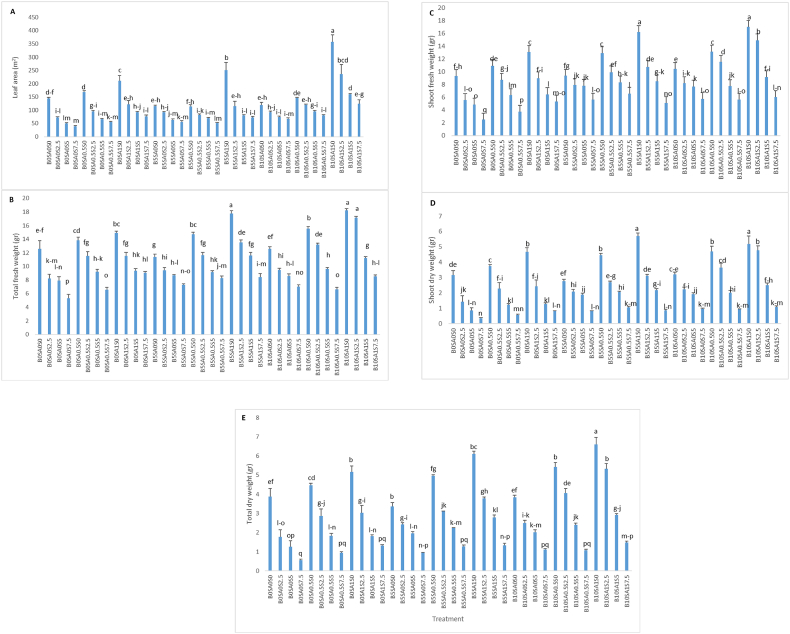
The interaction effect of biochar, salicylic acid and salt stress on morphological traits in soybean. S, SA and B are abbreviated of salt, salicylic acid and biochar. Fig. 3 continued. The interaction effect of biochar, salicylic acid and salt stress on morphological traits in soybean. S, SA and B are abbreviated of salt, salicylic acid and biochar Fig. 3 continued. The interaction effect of biochar, salicylic acid and salt stress on morphological traits in soybean. S, SA and B are abbreviated of salt, salicylic acid and biochar.

Moreover, the potency of salicylic acid and biochar usage simultaneously were exhibited in all studied treatments under salinity. For example, at 2.5 dS/m of salt stress, the utilization of 0, 0.5 and 1 mM of salicylic acid respectively increased the amount of leaf area (72.67-95.66–121.19 m^2^), total fresh weight (8.230-11.553–11.536 g), shoot fresh weight (5.547-8.738–8.951 g), shoot dry weight (1.435-2.288–2.439 g) and total dry weight (1.767-2.856–3.018 g), respectively (Fig. 3A–E). In addition, in salinity of 5 dS/m, the effect of salicylic acid (0, 0.5 and 1 mM) was completely obvious in such a way that leaf area, total fresh weight, shoot fresh weight, shoot dry weight and total dry weight were considered as (50.04-65.44–91.84 m^2^), (7.923-9.200–9.366 g), (4.861-6.321–6.468 g), (0.856-1.241–1.272 g), (1.255-1.823–1.808 g), respectively. Besides, at higher concentration of salinity (7.5 dS/m), various levels of salicylic acid as 0, 0.5 and 1 mM could affect significantly the leaf area (39.71-55.28–76.64 m^2^), total fresh weight (5.306-6.566–9.066 g), shoot fresh weight (2.487-3.736–5.310 g), shoot dry weight (0.344-0.581–0.817 g) and total dry weight (0.561-0.939–1.321 g), respectively (Fig. 3A–E).

### 3.3 Effect of biochar and salicylic acid on some physiological traits of soybean under salt stress

#### 3.3.1 Chlorophyll *a*

**Fig. 4:**
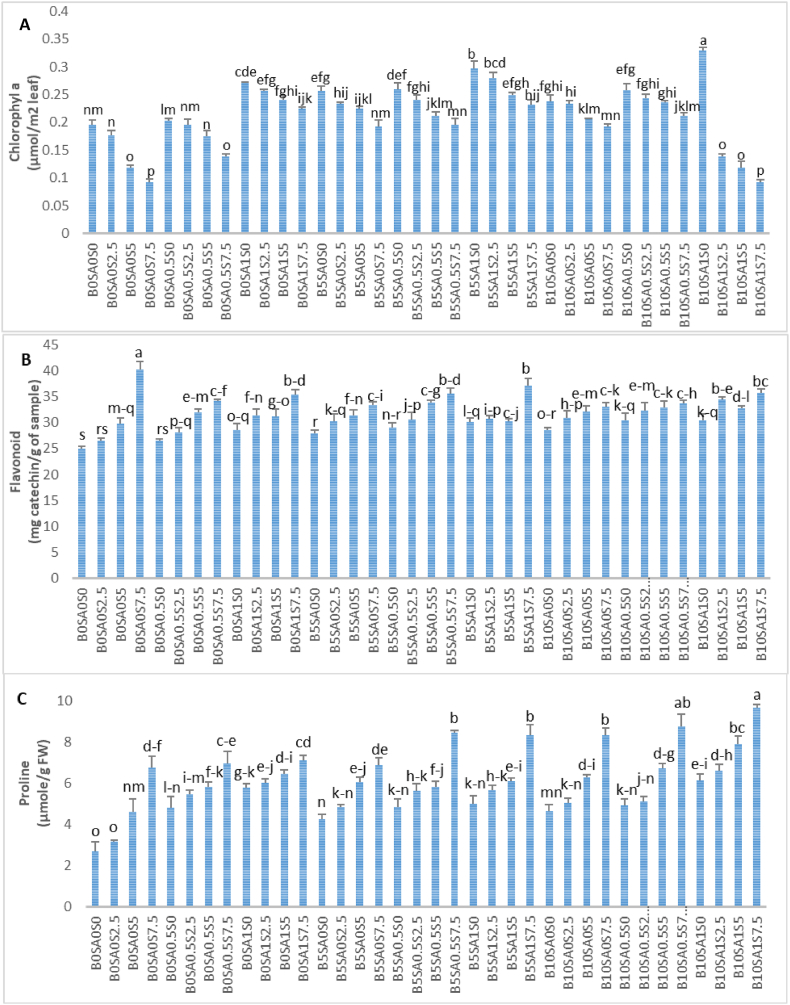
The interaction effect of biochar, salicylic acid and salt stress on some physiological traits in soybean. S, SA and B are abbreviated of salt, salicylic acid and biochar.

It was attained that salt stress led to decrease of chlorophyll *a* content in soybean, while singular or coupled application of biochar and salicylic acid resulted in increment of its amount. The maximum chlorophyll *a* content (0.329 mg/g FW) was observed by application of 10 WP of biochar and 1 mM of salicylic acid. However, the minimum of chlorophyll *a* content was 0.092 mg/g FW which was observed in the 7.5 dS/m of NaCl treatment and 7.5 dS/m of NaCl-10 WP of biochar and 1 mM of salicylic acid treatment combination (Fig. 4A).

Based on the results, with increasing biochar and salinity levels/salicylic acid and salinity concentrations, the amount of chlorophyll *a* indicated the decreasing trend, however, the combination of biochar and salicylic acid caused the increase of this trait under salinity regarding to the result that the interaction effect of 5 WP of biochar and 1 mM of salicylic acid showed the highest chlorophyll *a* (0.280 mg/g FW) under 2.5 dS/m of salinity among other similar treatments (Fig. 4A).

#### 3.3.2 Flavonoid and proline contents

According to the findings, the salt stress affected the total flavonoid content. Under salt stress, the total phenolic content was found to be increased (Fig. 4B). In detail, the total flavonoid content increased by 5.65, 16.05, and 35.02% with 2.5, 5, 7.5 dS/m of NaCl compared to control sample (Fig. 4B). Furthermore, the increasing effect of salicylic acid and biochar application simultaneously/separately on this parameter was shown in various levels of salinity. In 2.5 dS/m of salinity, flavonoid content increased 5.75 and 15.53% by application of B_0_SA_0.5_ and B_0_SA_1_, respectively. Also, the slight increase of flavonoid was observed 0.98 and 1.52% using B_5_SA_0.5_ and B_5_SA_1_, respectively, under 2.5 dS/m of salt stress. The increasing trend of flavonoid content was exhibited as 4.47 and 11.49% by 5 WP of biochar and foliar application of 0.5 mM of SA, respectively. Our obtained data showed that the lower amount of biochar led to minor change of flavonoid even in combination with salicylic acid. At 5 dS/m of salt stress, the usage of 0.5 mM of salicylic acid coupled with different concentrations of biochar expressed the more flavonoid content than application of 1 mM of SA. In higher concentration of salt (7.5 dS/m), the application of SA and biochar specified no reasonable effect on increment of flavonoid content (Fig. 4B). The enhancement trend of proline content was clearly observant by increase of salinity (Fig. 4C). According to the obtained data, the maximum and minimum proline content were 9.666 and 2.702 μmol/g FW by usage of B_10_SA_1_S_7.5_ and B_0_SA_0_S_0_ (control), respectively (Fig. 4C).

#### 3.3.3 Antioxidant enzyme activity

**Fig. 5:**
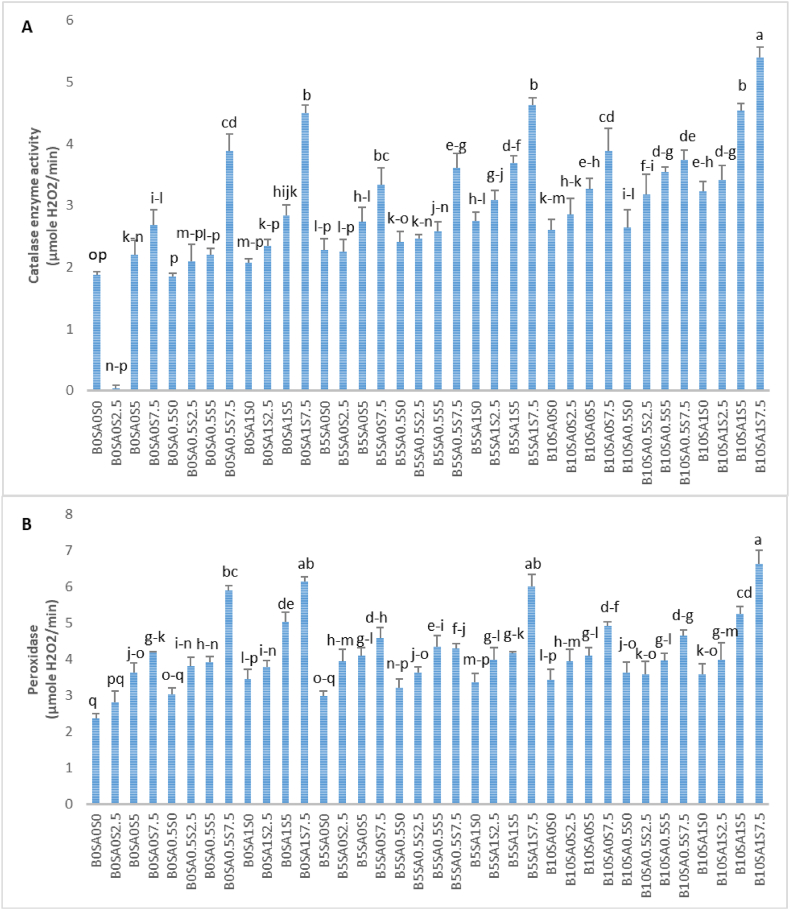
The interaction effect of biochar and salycilic acid on antioxidant enzyme activities (catalase and peroxidase) in soybean. S, SA and B are abbreviated of salt, salicylic acid and biochar.

Antioxidant enzyme activity plays an important role in the plant defense mechanism. In this experiment, CAT and POX activities were significantly increased in soybean that received NaCl. However, these activities were further augmented with SA and biochar as shown in Fig. 4. The highest and lowest CAT and POX activities were (5.40–6.63) and (1.88–2.35) μmole H_2_O_2_/min in treatments of B_10_SA_1_S^7.5^ and B_0_SA_0_S_0_, respectively (Fig. 5A and B). According to the obtained data, it is evident that the higher concentrations of biochar and salicylic acid had positive effect on these traits under various levels of salinity specially at higher level of NaCl like 7.5 dS/m (Fig. 5A and B).

## 4 Discussion

Salt stress is considered as one of crucial abiotic stresses which has detrimental effects on all developmental stages, including seed germination and post-germinative growth [32]. The toxic influences of salinity lead to oxidative stress following by ion toxicity which destruct several physiological practices in plants. Thus, accomplishment of practical and new approaches to prevent/reduce harmful sequels resulting from salt stress on morphological, physiological and biochemical properties would be of great importance [33].

In this experiment, a short cut approach was firstly used to identify salt tolerant genotype among various soybean genotypes at early growth stages. In second, the devastating effects of salinity on the candidate salt tolerant soybean genotype was examined by cost effective tools like exogenous spraying with salicylic acid and biochar application to soil under salinity.

### 4.1 Impact of salt stress on soybean seed germination

The rate and percentage of seed germination are one of main factors which suffered from salinity at early growth stages [34]. According to the results, with increasing salinity, the all studied traits related to soybean seed germination like root and shoot length, root and shoot dry weight, total dry weight and seed germination rate indicated significant reduction (Table 1). The obtained data was in parallel with the results reported on soybean [35], sorghum [36], melon [37], and common bean [38]. It could be suggested that the adverse effects of salinity on cell division and elongation might result in reduction of rootlet and shootlet growth and root and shoot biomass [39]. Moreover, decrease in biomass production could be resulted from nutrient imbalance, ROS overproduction and enzymatic activities inhibition which effects on cellular components and biological cell membrane [40].

In addition, the present data showed that different soybean genotypes showed various reactions exposed to different salinity levels (Table 1). It was expressed that the easiest and effective method to manage and control the side effects of salt stress is to utilization of salt tolerant cultivars since a plant species with different cultivars may display various ranges of tolerance to salinity factors [41,42].

In the present experiment, rootlet length was more affected by salinity than shootlet length which could be referred to increase of root biomass by salt stress [43]. Since rootlets are the first organ exposed to the soil and following by salinity, they are firstly subjected to secondary osmotic stress which needs higher energy and metabolites to deactivate the detrimental ions of salinity [44]. Furthermore, the reduction of dry and fresh weight of rootlet and shootlet caused by salinity can be referred to decrease of amount of primary water and its absorption rate as well as the negative effect of low osmotic potential and ion toxicity on biochemical processes in early stages of seed germination [45]. It was reported that the ion accumulation resulted from salinity may bear drought stress as well which lead to decrease of water absorption by plant tissues, cell growth and development accompanying by reduction in rootlet and shootlet growth [46].

### 4.2 Growth improvement due to SA and biochar application under salinity

The results showed a negative relationship between various levels of salinity stress and growth parameters including leaf area, shoot dry/fresh weight, and total fresh/dry weight (Fig. 3). In general, plant growth is affected by salt stress through 1) decrease of water potential in soil and 2) ionic effects in metabolisms processes [47]. One of the most sensible influences of decreased growth is the reduced leaf area which cause reduction in photosynthesis rate in whole plant even without decline of photosynthesis rate per plant. Besides, it was noted that the decrease in dry weight of aerial part of plant in salinity condition is accompanied by decrease in leaf area and photosynthesis rate [48]. This reduction is probably due to harmful effects of salinity stress on rate of growth and reduction of photosynthetic level which can reduce total dry matter in plant. Meanwhile, a part of the produced materials is used to provide osmotic conditions needed by plant [49].

However, our data indicated that the interaction effect of SA and biochar separately/copuled plays major roles in regulating all aforementioned traits which were reduced by different levels of salt stress (Fig. 2). In addition, the increasing effect of 1 mM salicylic acid in all treatments of biochar was observed on all morphological traits under salinity in Fig. 2. In similar studies, the efficacious foliar application of SA on the traits studied was investigated by Refs. [[50], [51], [52]] which was in agreement with our obtained data.

Additionally, the outcome of the present study exhibited that biochar had positive effect on shoot biomass and total dry and fresh weight which was compatible with the resultant data on maize [53], potatoes [54], and tomato [55]. Overall, it was proved that biochar could alleviate the adverse impact of salinity stress on plants [56]. In fact, soil treated with biochar have the potential to increase/improve plant growth and biomass under saline conditions. Although the potential effect of biochar was lower than SA on all morphological parameters in the current research, it could be noticed that the simultaneous application of 10 WP of biochar and 1 mM of salicylic acid was the most appropriate treatment to improve morphological properties in soybean at different levels of salinity (Fig. 3).

### 4.3 The interaction effect of salicylic acid, biochar and salinity on physiological properties

#### 4.3.1 Chlorophyll *a* content

Salt stress may cause senescence and reduced photosynthesis activity which consequences from decreased chlorophyll content [57]. In our case, the interaction effect of SA, biochar and Salt was significant for chlorophyll *a* content (Fig. 4A). The deleterious effect of salt stress led to decrease in chlorophyll *a* content (Fig. 4A). Different researches have been examined the influences of salinity on photosynthesis activity [58,59]. It was stated that salinity diminished gas exchange, stomatal conductance, electron transport chain, and thus chlorophyll synthesis in plants. This reduction may result from degradation of chlorophyll structure because of ROS production and substitution of essential elements by Cl^−^ and Na^+^ [60].

It was obvious from our findings that the coupled application of 5 WP of biochar and 1 mM of salicylic acid showed the highest increment of 64.71%, 110%, 152% of chlorophyll *a* content at 2.5, 5 and 7.5 dS/m, respectively compared to utilization of no biochar and salicylic acid (Fig. 4A). Previous studies have been performed the capability of biochar to enhance phothosysnthetic activity in plants. It was demonstrated that higher concentration of biochar could increase photosynthetic rate in maize which probably because of Na^+^ content decline followed by an increase in chloroplast ultrastructure [61,62]. In another study, it was reported an increase of chlorophyll content in maize leaves resulting from increase of CO_2_ uptake through interaction effect of biochar and salinity [63]. In addition, wheat salt-stressed plants were positively influenced by biochar supplementation to decline sodium accumulation [64]. Besides of biochar, SA can be able to increment photosynthetic pigment content by uptake and transport nutrients and protecting them by alleviation salt stress inhibition of photosynthetic parameters [65].

#### 4.3.2 Flavonoid content

One of defensive mechanisms in plants have been attributed to secondary metabolites like phenolic compounds, in which flavonoid plays important role of antioxidant activity and free radical scavenging [66]. In fact, flavonoid belongs to non-enzymatic antioxidants, contribute significantly as scavenging free radicals accumulating in various tissues in plants to protect plant from salt stress [67]. The present study showed the increment trend of flavonoid content by increasing salt stress levels (Fig. 4B). This result is similar to previous results which were reported on *Thymus vulgaris* L. [68], *Hibiscus cannabinus* L. [69], and *Aegilops cylindrica* [70]. In point of fact, numerous researchers have consistently reported on the powerful relationship between polyphenols and abiotic stress tolerance which can be known as excellent indicator of conservancy of redox condition in plant cells [71]. However, usage of flavonoid enhancer might be the easiest and applicable tools to conserve and protect plant from abiotic stress such as salinity. In this case, this experiment sought to illustrate the defensive role of salicylic acid and biochar and its combined application in alleviating salt stress damaging effects in soybean. On the basis of obtained data, the protective role of salicylic acid and biochar was entirely evident, since the maximum content of flavonoid were 34.44 mg/g (under 2.5 dS/m of NaCl by application of 10 WP biochar and 1 mM of SA) and 33.91 mg/g (under 5 dS/m of NaCl by application of 5 WP of biochar and 0.5 mM of SA). The similar results were gained by Ref. [72] reported on the application of SA on *Panax ginseng* which caused remarkable increase of flavonoid content. Although, few studies have been performed to evaluate the coincidence utilization of biochar and SA, it has been proved the effectiveness of biochar to improve flavonoid content in plants under salinity [73,74].

Moreover, our findings stipulated that foliar application of salicylic acid and biochar application to soil couldn't play successive and effective role to increase flavonoid content in order to make soybean plant more tolerant to salinity stress at 7.5 ds/m of NaCl. This was probably related to severe susceptibility of soybean to higher concentration of NaCl. Due to the fact that, soybean is classified as a moderately salt-tolerant crop and the final yield of soybean will be reduced when soil salinity exceeds 5 dS/m [75].

#### 4.3.3 Proline content

Numerous studies have been proved the role of proline as a primary defense response to maintain osmotic pressure in cells of salt stressed plants [76]. Indeed, accumulation of proline can adjust osmotic pressure, stabilize membrane and detoxifies injurious factors in plants subjected to salinity conditions [77]. It was obtained from the present study that proline content increased by increment of salinity stress. In addition, an increment of proline content was observed with singular and coupled application of salicylic acid and biochar. The highest amount of proline was gained as 6.596, 7.920 and 9.666 μmol/g FW under 2.5, 5 and 7.5 dS/m of NaCl, respectively by utilization of 10 WP of biochar and 1 mM of SA (Fig. 4C). As a matter of fact, proline accumulation in plant tissues is the most common induced alteration arising from salt stress. This accumulation might be attributed to decrease of proline oxidation, its synthesis induction from glutamate and increase of protease activity [78]. Furthermore, SA has known as intracellular enhancer of proline production to protect plant from salinity condition. With respect to former studies, it was exhibited that proline content enhanced in response to NaCl and/or SA treatment emphasizing the additive effect of their interaction [79]. In other study, it was reported that SA improved salt tolerance capacity of *Saponaria officinalis* by incrementing proline content and photosynthesis rate. Besides, few examinations have been performed to evaluate biochar effects on proline content in which their results were differed from the data from this survey. It was suggested the reduction of CAT and SOD activity, proline, sucrose, H_2_O_2_, and MDA contents in leaf of cabbage seedling by interaction effect of biochar and salinity which was not in agreement with our data [80]. While, it was showed that biochar could mitigate drought stress by increase of 91% proline content in soybean leaves [81]. The differences between biochar behaviors may due to the reason that some biochar has the capability of osmotic potential reduction by accumulation osmolytes like proline to conserve water potential [82,83]. And some other biochar develop moisture maintenance ability of the soil by generation of heterogenous pore ranges in the soil [84].

#### 4.3.4 Antioxidant enzyme activity

Antioxidant enzymes like catalase and peroxidase are the important key factors for scavenging ROS under abiotic stresses [85]. The defensive way of catalase is to convert photo-respiratory or respiratory H_2_O_2_ into water and peroxidase is able to oxidase co-substrates molecules such as phenolic compounds and/or antioxidants to decompose H_2_O_2_ [86]. The present results expressed the increasing trend of antioxidant enzyme activities by increasing salinity levels, while application of biochar and salicylic acid were shown as enhancer/helper to diminish negative effects of ROS under salinity (Fig. 5). Previous reports on application of biochar confirmed our data by mentioning this issue that biochar can boost antioxidant enzymes and impair ROS production in leaves by reduction of oxidative stress [87]. In addition, in another studies, it was suggested that biochar has the capability of stimulating antioxidants to scavenge ROS produced by salt through improving cellular redox homeostasis which cause reinforcement of salt tolerant [88,89]. In line with the previous reports, it was observed that SA augmented SOD, CAT and POX activities under salinity [90]. Also, the proficiency of SA on SOD, POX, and CAT performance were previously proved in pistachio by Refs. [91,92].

In the concrete, little information might be accessible on the interaction effect of biochar and salicylic acid on activities of antioxidant enzymes under salt stress in soybean. Based on this issue, the data resulted from this research can supply suitable information on the protective role of antioxidants against reactive oxygen species (ROS) under conditions of non-biochar and/salicylic acid application and application both of them under salinity stress.

## 5 Conclusion

**Fig. 6:**
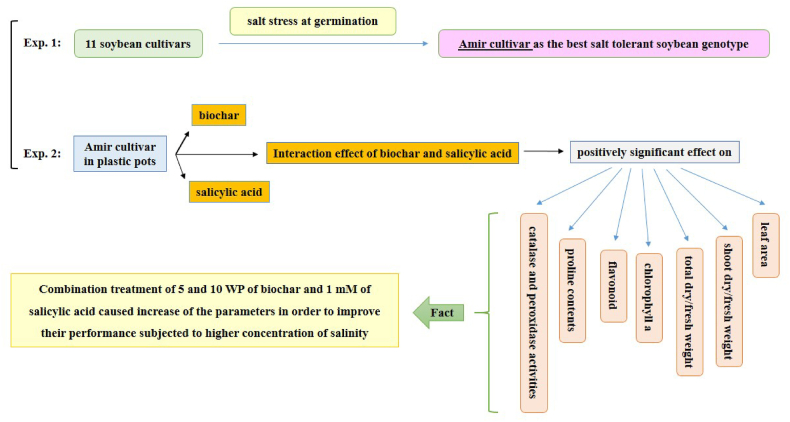
The summary of procedure and resultant data obtained from the interaction effect of biochar and salicylic acid on some soybean traits under different concentrations of salinity.

In the present study, salt stress showed the negative effects on all seed germination traits among soybean cultivars and their reactions exposed to salt stress varied resulting Amir genotype was selected the most appropriate soybean cultivar in relation to tolerance to salt stress. In the following results, we explored, for the first time, the interaction effect of salicylic acid and biochar on the selected soybean genotype under salt stress. The data of this study clearly revealed that soybean suffered from all morpho-physiological traits. However, different levels of SA and biochar could increase 1) the contents of morphological traits such as shoot fresh/dry weight, total dry/fresh weight and leaf are and 2) physiological properties including photosynthesis pigment (chla), proline content, flavonoid content and antioxidant enzyme activities (CAT and POX). In fact, the simultaneous application of biochar (5 and 10 WP) and salicylic acid (1 mM) were indicated as the most effectual strategy to lessen disastrous consequences of salt stress in soybean. The obtained results can be used for further sustainable soybean and other crop productivity studies along with other morpho-physiology parameters and different levels of biochar and salicylic acid elicitor. For more and easier perception, the summary of data obtained from this study was presented in Fig. 6.

## Funding statement

There was no source of funding for the manuscript.

## Data availability statement

Data included in article/supp. material/referenced in article.

## CRediT authorship contribution statement

**Mohammad Mehdi Alizadeh:** Writing – original draft. **Mahyar Gerami:** Supervision. **Parastoo Majidian:** Writing – review & editing, Writing – original draft, Visualization, Validation, Supervision, Software, Project administration, Methodology, Investigation, Formal analysis, Data curation, Conceptualization. **Hamid Reza Ghorbani:** Software, Project administration, Methodology, Investigation, Formal analysis, Data curation, Conceptualization.

## Declaration of competing interest

The authors declare that they have no known competing financial interests or personal relationships that could have appeared to influence the work reported in this paper.
